# DCDW-YOLOv11: An Intelligent Defect-Detection Method for Key Transmission-Line Equipment

**DOI:** 10.3390/s26031029

**Published:** 2026-02-04

**Authors:** Dezhi Wang, Riqing Song, Minghui Liu, Xingqian Wang, Chengyu Zhang, Ziang Wang, Dongxue Zhao

**Affiliations:** 1 State Grid Liaoning Electric Power Co., Ltd., Dandong 118000, China; 2 College of Information and Electrical Engineering, Shenyang Agricultural University, Shenyang 110016, China; 3 National Digital Agriculture Regional Innovation Sub-Center, Shenyang 110866, China; 4 Liaoning Engineering Research Center for Information Technology in Agriculture, Shenyang 110866, China

**Keywords:** key equipment of transmission lines, deep learning, YOLOv11, unmanned aerial vehicle (UAV), object detection

## Abstract

The detection of defects in key transmission-line equipment under complex environments often suffers from insufficient accuracy and reliability due to background interference and multi-scale feature variations. To address this issue, this paper proposes an improved defect detection model based on YOLOv11, named DCDW-YOLOv11. The model introduces deformable convolution C2f_DCNv3 in the backbone network to enhance adaptability to geometric deformations of targets, and incorporates the convolutional block attention module (CBAM) to highlight defect features while suppressing background interference. In the detection head, a dynamic head structure (DyHead) is adopted to achieve cross-layer multi-scale feature fusion and collaborative perception, along with the WIoU loss function to optimize bounding box regression and sample weight allocation. Experimental results demonstrate that on the transmission-line equipment defect dataset, DCDW-YOLOv11 achieves an accuracy, recall, and mAP of 94.4%, 92.8%, and 96.3%, respectively, representing improvements of 2.8%, 7.0%, and 4.4% over the original YOLOv11, and outperforming other mainstream detection models. The proposed method can provide high-precision and highly reliable defect detection support for intelligent inspection of transmission lines in complex scenarios.

## 1. Introduction

As a vital element of national infrastructure, the secure and reliable functioning of power systems is essential for sustained socioeconomic growth. Overhead transmission lines represent the main means of electricity delivery, and their operating status has a direct influence on the overall safety and reliability of power system operation [1,2,3]. However, transmission lines are long-term exposed to complex outdoor environments and are frequently subjected to extreme weather conditions, such as high temperatures, strong winds, rain and snow, and lightning. Under these adverse conditions, various structural defects, including insulator damage, pin loss, and conductor strand breakage, are prone to occur [4,5]. If such defects cannot be detected and addressed in a timely manner, they may lead to localized equipment failures or even large-scale power outages, thereby posing serious threats to power system stability and public safety [6,7].

In routine operation and maintenance activities, conventional approaches for inspecting transmission lines primarily rely on ground-based manual patrols and helicopter-assisted inspections [8,9]. Manual inspection typically relies on trained personnel to conduct close-range observation and photographic recording by walking along the lines or using ground transportation. This approach is labor-intensive and inefficient, and its effectiveness is further limited by terrain complexity and adverse weather conditions, making it difficult to achieve comprehensive coverage and accurate identification of elevated targets.

Beyond manual and aerial visual inspection, early automated defect-detection studies mainly relied on traditional image-processing and machine learning techniques. Typical methods include edge detection, threshold segmentation, texture analysis, and handcrafted feature descriptors such as Histogram of Oriented Gradients (HOG), Local Binary Patterns (LBP), and color- or shape-based features, followed by conventional classifiers such as support vector machines (SVMs), k-nearest neighbors (k-NN), and random forests. These methods have been applied to specific inspection tasks, such as insulator contamination detection or conductor surface analysis, under relatively controlled imaging conditions. However, their performance is highly sensitive to illumination variations, background clutter, viewpoint changes, and scale diversity, which are common in UAV-based transmission-line inspection scenarios. As a result, traditional approaches often suffer from limited robustness and poor generalization when deployed in complex outdoor environments.

Beyond visual inspection-oriented studies, fault diagnosis research in other industrial domains has emphasized discriminative feature representation and system-level modeling. For example, Ji et al. [10] proposed an Extended Shapelet Learning-based Discriminant Dictionary framework for froth flotation fault recognition, in which spatio-temporal shapelet features and discriminative dictionary learning were jointly employed to enhance class separability and robustness. Although this approach targets industrial process monitoring rather than transmission-line inspection, it highlights the importance of coordinated feature modeling and robustness-oriented system design, which is also highly relevant to complex multi-defect detection scenarios in power systems.

In recent years, the rapid progress of unmanned aerial vehicle (UAV) technology has led to UAV-based inspections becoming a promising alternative to traditional manual and helicopter methods, owing to their lower operational costs, greater flexibility, and ease of deployment. By mounting high-resolution imaging sensors on UAV platforms, transmission lines can be rapidly inspected from multiple altitudes and viewing angles, enabling the acquisition of clear image data for subsequent defect identification through image processing techniques. Compared with traditional inspection methods, UAV-based inspection significantly improves inspection efficiency and task throughput while reducing labor and equipment costs [11]. It is particularly suitable for inspections in complex terrain or high-risk areas and has therefore become a key approach for intelligent transmission-line inspection [12,13].

Recent progress in deep learning has enabled UAV inspection images to be exploited more effectively for transmission-line condition assessment. In this context, object detection is particularly attractive because it yields both semantic labels and spatial localization, which can be directly integrated into downstream procedures such as defect reporting and maintenance prioritization. By replacing labor-intensive visual screening with model-based inference, automated detection can increase inspection throughput and consistency while maintaining reliable localization performance. Therefore, designing defect detectors that balance accuracy, computational efficiency, and ease of deployment remains an important practical objective for safeguarding power-system operation [14].

Deep learning-based detectors are commonly implemented following either a proposal-driven pipeline or a single-pass paradigm. Proposal-driven (two-stage) approaches, including R-CNN, Faster R-CNN, and Mask R-CNN [15,16,17], first identify candidate regions and then refine each candidate through classification and bounding-box regression. Although two-stage detectors achieve high accuracy on many benchmarks, their reliance on additional proposal generation and multi-step feature processing often introduces considerable computational overhead, limiting inference speed and making them less ideal for real-time or resource-limited applications. In contrast, single-stage detectors, such as the YOLO series [18,19] and SSD [20], perform end-to-end prediction of object categories and locations through a simplified inference pipeline, rendering them more suitable for scenarios where low latency and deployment efficiency are essential.

In the context of transmission-line inspection, single-stage detectors have attracted increasing attention due to their favorable trade-off between detection accuracy and inference speed. Building on this line of research, Du et al. [21] proposed YOLOv5-nS for insulator pin detection, achieving substantial reductions in parameters and model size while improving the frame rate under comparable accuracy. Han et al. [22] introduced local cross-channel interaction into a YOLOv5-based framework to reduce computation, although modeling of long-range channel dependencies may be limited. Hu et al. [23] improved YOLOv8 for insulator defect detection by adopting a deformable-attention backbone constructed from Deformable ConvNets v2 modules and incorporating global attention. Wu et al. [24] enhanced YOLOv8 with a GSConv-based adaptive-threshold strategy and a lightweight Slim-Neck design to detect conductor damage in transmission-line imagery.

More recently, Ji et al. [25] introduced an enhanced YOLOv11 model that incorporates adaptive feature fusion, attention mechanisms, and a lightweight network design. This approach substantially improves the accuracy of insulator defect detection while reducing model complexity, making it more feasible for deployment on devices with limited computational resources. Zhao et al. [26] presented a lightweight improvement strategy based on YOLOv11n, in which multidimensional dynamic convolution (ODConv) was employed to reconstruct the C3 module, the SlimNeck structure was adopted to reduce computational complexity, and the WIoU loss function was used to optimize the training process. The proposed method achieved superior detection accuracy and recall compared with YOLOv8 and YOLOv10 while maintaining low computational overhead, providing an effective solution for real-time and high-precision defect detection of transmission-line insulators.

However, most existing studies primarily focus on the detection of a single type of defect, while research on the joint detection of multiple defect categories in transmission lines remains relatively limited. In related research, Peng et al. [27] proposed the EDF-YOLOv5 method based on YOLOv5s, in which the EN-SPPFCSPC module was integrated to enhance feature extraction for small defect targets, the DCNv3C3 structure was introduced to improve adaptability to irregular defects, and the Focal-CIoU loss function was employed to emphasize high-quality sample training, thereby improving detection performance and generalization ability. Nevertheless, this method still exhibits limitations when dealing with low image quality and complex background interference. Wang et al. [28] proposed an enhanced detection method built upon the Faster R-CNN framework. By adopting MobileNet as the backbone to reduce computational burden, employing soft-NMS to handle occluded targets, and introducing context-aware ROI pooling to preserve small-target details, the detection accuracy and reliability were effectively enhanced. In addition, Kalman filtering was applied to further refine the detection results. However, the two-stage design leads to a relatively long inference pipeline, making it less suitable for UAV-based online inspection tasks that require low latency.

Motivated by the above limitations and the practical demand for accurate multi-class defect detection under complex transmission-line environments, this study proposes DCDW-YOLOv11, a task-oriented and system-level optimized detection framework built upon the YOLOv11 architecture. Unlike existing works that focus on a single defect type or rely on computationally expensive two-stage pipelines, the proposed method aims to achieve a balanced improvement in detection accuracy, robustness, and deployment efficiency for UAV-based transmission-line inspection. It should be emphasized that this work does not introduce fundamentally new algorithmic components; instead, it focuses on the coordinated integration, adaptation, and validation of established techniques to address application-specific challenges in transmission-line inspection scenarios.

Specifically, the contributions of this work can be summarized as follows:

(1) A task-adapted backbone optimization strategy is constructed by introducing multidimensional dynamic convolution (ODConv) and attention mechanisms, which adaptively adjust convolutional weights to better capture discriminative features of small and irregular defect targets, thereby improving feature representation under complex backgrounds.
(2) The DyHead detection head is incorporated to enable dynamic multi-scale feature fusion across spatial, channel, and task dimensions, forming an application-oriented multi-scale perception mechanism that enhances detection consistency for defects with large scale variations across different defect categories.
(3) The WIoU loss function is adopted to optimize bounding-box regression by dynamically reweighting samples according to localization quality, which serves as a training optimization strategy to alleviate the negative impact of low-quality samples and improve localization accuracy for small and partially occluded defects.
(4) A unified and lightweight engineering framework is constructed for joint detection of multiple transmission-line defect types, including conductor strand breakage and pin loss, demonstrating stable performance across different defect forms through coordinated module integration rather than algorithmic novelty, while avoiding excessive computational overhead.

Through these coordinated improvements, DCDW-YOLOv11 achieves superior detection accuracy and robustness while maintaining low model complexity, making it well suited for real-time UAV inspection scenarios and providing a practically deployable, application-driven solution for intelligent condition monitoring of transmission-line infrastructure.

## 2. Materials and Methods

### 2.1. Dataset Construction

In this study, a dedicated dataset was constructed for defect detection of critical transmission-line equipment under complex environmental conditions. The dataset was collected in multiple regions of Liaoning Province, China, including Shenyang, Dandong, and Anshan, which feature diverse geographical characteristics and inspection environments. The dataset was collected through a collaborative effort between the research team and affiliated units of the State Grid Corporation, yielding a total of 6000 raw images. All images were acquired during actual transmission-line inspection operations using UAVs equipped with digital cameras, ensuring high field representativeness and diverse scene coverage.

**Figure 1:**
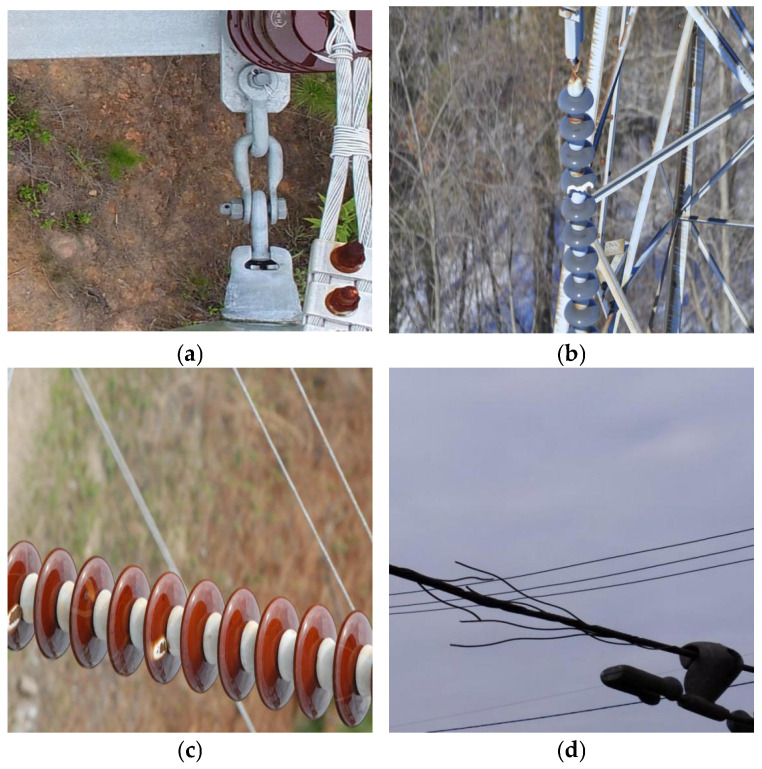
Defect data of key equipment in transmission lines. (**a**) DefectPin. (**b**) Damaged. (**c**) Flashover. (**d**) Sangu.

To improve the quality and usability of the dataset, a systematic data cleaning process was performed. This process involved removing duplicate images and discarding samples with severe blur, overexposure, or significant occlusion, thereby substantially enhancing image clarity and target distinguishability. Owing to variations in terrain conditions, background complexity, inspection angles, and illumination across different cities, the collected images exhibit substantial diversity in scene appearance and defect presentation. This diversity increases the difficulty of defect detection and provides a more realistic evaluation setting that partially reflects variations encountered in real-world transmission-line inspection scenarios. During the annotation stage, defect targets in each image were meticulously labeled using the professional annotation tool LabelImg. As a result, a high-quality dataset comprising 5268 images was established, covering four categories of defects related to critical transmission-line equipment. Detailed statistical information of the dataset is provided in Table 1, and representative examples of typical defect samples are illustrated in Figure 1.

For model training and performance evaluation, the constructed dataset was split into a training set and a validation set with a ratio of 8:2, comprising 4214 images for training and 1054 images for validation. It should be noted that the class distribution of the dataset is inherently imbalanced, which is consistent with real-world inspection statistics where certain defect types occur more frequently. This imbalance was addressed through data augmentation and loss optimization during training, and per-class detection performance was reported to ensure transparent and reliable evaluation.

### 2.2. YOLOv11 Algorithm

The YOLO (You Only Look Once) family has been iteratively advanced over recent years, and YOLOv11 is regarded as one of the most recent variants in this line of research [29,30,31]. Relative to earlier releases, YOLOv11 provides a favorable trade-off among detection accuracy, runtime speed, and computational cost. Notably, it can preserve competitive detection performance while reducing parameter redundancy, which contributes to improved generalization across different scenarios. Benefiting from this balance between effectiveness and efficiency, YOLOv11 has been adopted in a broad range of applications, including industrial inspection, medical image analysis, and security monitoring, and it is also suitable for challenging visual tasks such as defect detection in overhead transmission-line inspection.

**Figure 2:**
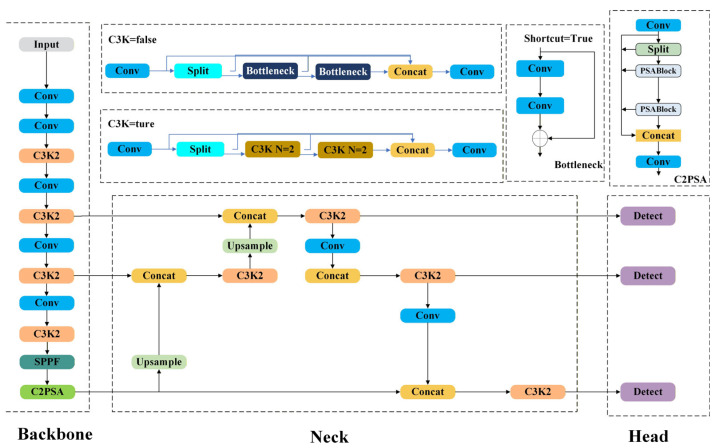
YOLOv11 network structure.

As shown in Figure 2, the YOLOv11 framework is typically organized into four modules: an input stage, a backbone, a neck, and a detection head [32]. The input stage conducts adaptive image resizing and normalization/alignment of data distribution to better match the training setting. The backbone then extracts multi-level feature representations from the processed images and delivers semantic cues to the neck and detection head, which subsequently perform object localization and classification.

Compared with earlier versions such as YOLOv8, YOLOv11 introduces the C3k2 module into the backbone network. Built upon the original C2f structure, this module optimizes feature extraction paths and gradient propagation mechanisms, effectively combining the advantages of C3 and C2f to improve feature extraction and transmission efficiency. In addition, a C2-PSA module is appended after the SPPF layer, enhancing the model’s adaptability to occluded targets and multi-scale features.

For feature fusion, YOLOv11 mitigates information attenuation in deep networks by optimizing gradient paths and integrates modules such as C3k2, upsampling, convolution, and concatenation within the neck network [29], thereby enriching multi-level feature representations and improving both detection performance and efficiency. Furthermore, YOLOv11 inherits the PGI framework from YOLOv9 and retains the dual-label assignment mechanism of YOLOv10, which simplifies the training-to-deployment pipeline and further enhances real-time performance and deployability.

However, directly applying YOLOv11 to UAV-based power line inspection scenarios still presents several challenges. Defects in transmission lines are typically characterized by small target sizes and complex background interference, which makes existing visual detection frameworks prone to missed detections and false positives. In addition, the model still exhibits limitations in multi-scale feature fusion, as the integration between shallow positional information and deep semantic features remains insufficient. Consequently, there is considerable room for improvement in fine-grained defect perception and classification performance.

### 2.3. DCDW-YOLOv11 Model

Compared with the official YOLOv11 architecture, the proposed DCDW-YOLOv11 introduces a set of task-oriented structural modifications at the backbone, detection head, and optimization levels, specifically tailored for UAV-based transmission-line defect inspection. While the original YOLOv11 primarily adopts standard convolutional blocks and a static detection head, DCDW-YOLOv11 enhances feature adaptability and robustness by incorporating deformable convolution, attention mechanisms, dynamic feature aggregation, and a localization-aware loss function. These modifications preserve the original one-stage detection paradigm of YOLOv11, while improving its suitability for detecting small, irregular defects under complex background conditions.

**Figure 3:**
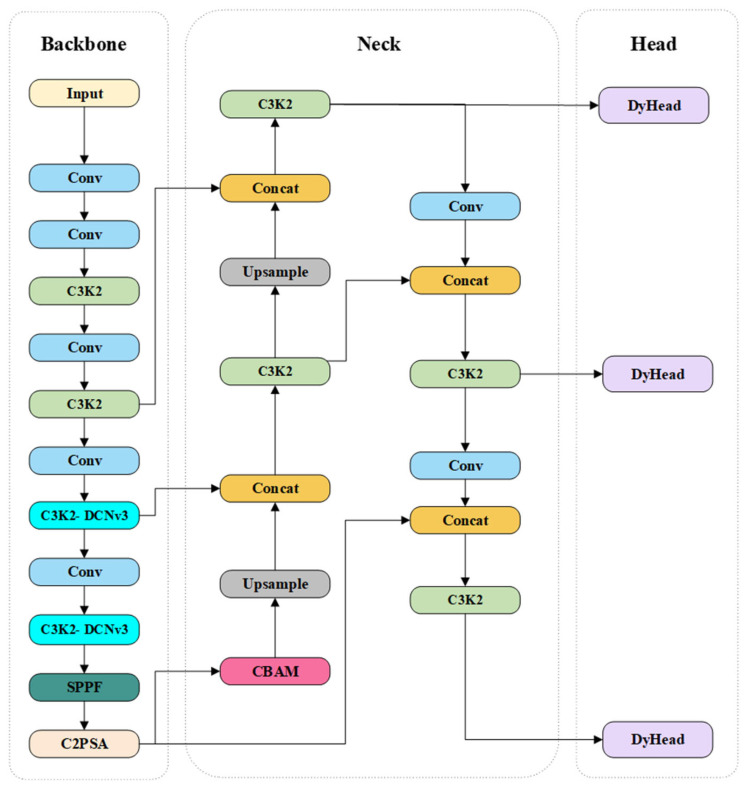
DCDW-YOLOv11 Network Structure.

To address the challenges commonly encountered in UAV-based power line inspection—such as feature information loss, limited detection accuracy for small targets, and high false and missed detection rates—four targeted optimization strategies are introduced in DCDW-YOLOv11. First, a C3k2-DCNv3 module is integrated into the backbone network. By leveraging the dynamic modeling capability of deformable convolutions, this module enhances the representation of geometric structures and dense small-target features, thereby improving the recognition accuracy of key components such as conductor strand breakage, insulators, and pins under complex backgrounds. Second, the convolutional block attention module (CBAM) is embedded to apply dual attention weighting along the channel and spatial dimensions, enabling the network to focus on salient defect regions while suppressing background interference. Third, the original detection head is replaced with a DyHead-based detection head. Through cross-level and multi-dimensional attention mechanisms, DyHead strengthens the model’s scale, spatial, and task awareness, resulting in improved detection performance for multi-scale targets, particularly small objects. Finally, the WIoU loss function is adopted for bounding box regression. By dynamically reweighting training samples according to localization quality, WIoU alleviates the influence of low-quality samples and improves localization accuracy. The overall architecture of the proposed DCDW-YOLOv11 model is illustrated in Figure 3.

#### 2.3.1. C3K2- DCNv3

**Figure 4:**
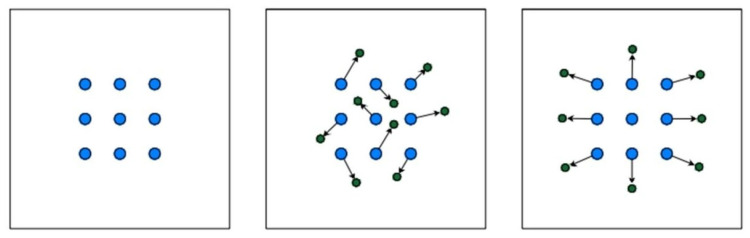
Variable convolution pixel point sampling.

Defects in key components of transmission lines typically exhibit small object sizes, diverse appearances, and irregular structures. The standard convolution employed in YOLOv11 has inherent limitations when dealing with such targets that involve geometric deformation or local structural variations. Since convolution kernels sample features at fixed grid locations, they lack the flexibility to adapt to variations in scale, pose, and shape across different spatial positions, which may lead to suboptimal feature representation. Although atrous convolution can enlarge the receptive field to capture broader contextual information, its sampling pattern remains fixed, limiting its ability to model non-rigid deformations effectively. By contrast, deformable convolution incorporates learnable offset parameters that allow convolution kernels to adaptively adjust their sampling locations in response to input features. This adaptive sampling strategy enhances the network’s capability to model non-rigid object deformations, partial occlusions, and complex background interference, which are commonly encountered in UAV-based transmission-line inspection scenarios [27]. As illustrated in Figure 4, deformable convolution dynamically modifies its sampling positions to better align with the actual geometric structure of the target. This capability enhances the model’s ability to extract and represent discriminative features from small-scale and irregular defects, thereby improving detection performance in complex transmission-line environments.

**Figure 5:**
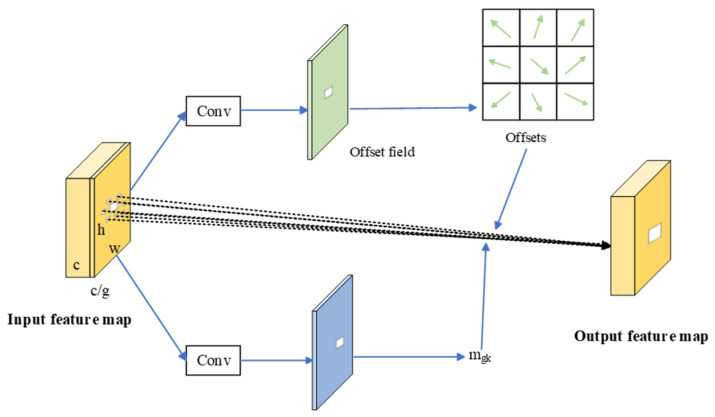
The structure of the DCNv3 module.

DCNv3 (Deformable Convolution Network v3) employed in this study [33] extends DCNv2 through several architectural updates. Instead of performing deformation modeling and feature mapping within a single convolution, DCNv3 decouples these roles by introducing depthwise modulation for spatially adaptive sampling and pointwise projection for channel mixing, which improves sampling flexibility without sacrificing efficiency. Moreover, the spatial aggregation is organized in a multi-group manner, where different groups maintain independent offsets and modulation factors, allowing the operator to accommodate a wider range of geometric variations and yielding more robust representations for irregular and small-scale targets. DCNv3 also modifies the normalization scheme by applying a Softmax over the sampling locations (rather than a Sigmoid-based formulation), which tends to provide smoother gradients and more stable optimization. The architecture of DCNv3 is presented in Figure 5, and its mathematical definition is given in Equation (1).

(1) $$y\left(p_{0}\right)=\Sigma_{g=1}^{G}\Sigma_{k=1}^{K}w_{g}m_{gk}x_{g}\left(p_{0}+p_{k}+\Delta p_{gk}\right)$$

where  G denotes the number of convolution groups; $w_{g}$ represents the shared projection weights of the gth group; $m_{gk}$ refers to the normalized modulation factor associated with the kth sampling point in the g-th group; $x_{g}$ denotes the sliced input feature map for group *g*; and $\Delta p_{gk}$ corresponds to the offset of the sampling locationin the g-th group.

#### 2.3.2. CBAM

**Figure 6:**
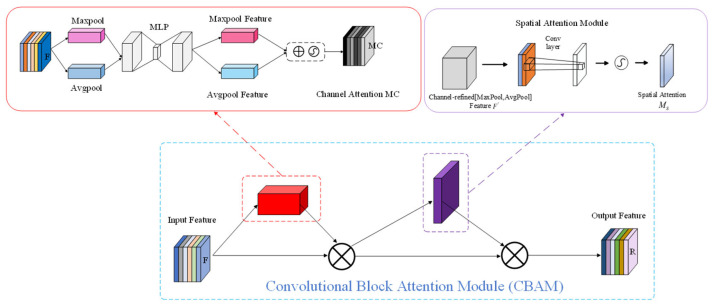
The schematic diagram of CBAM attention mechanism.

Conventional feature representation often struggles to capture the pronounced scale changes and spatial variability exhibited by defects on key transmission-line components. To improve feature separability in cluttered scenes, we integrate the convolutional block attention module (CBAM) into the network. CBAM improves feature representation by adaptively reweighting responses along both channel and spatial dimensions, which enhances detection accuracy and model robustness [34]. The module consists of a channel attention stage followed by a spatial attention stage. Each stage produces an attention map—channel-wise and spatial-wise, respectively—and the resulting weights are used to rescale the intermediate features via element-wise multiplication. In this way, CBAM can attenuate background-related activations while strengthening defect-relevant cues, leading to more informative representations for targets with diverse structures and sizes. The structure and computation pipeline of CBAM are presented in Figure 6.

CBAM begins feature recalibration with the Channel Attention Module (CAM), which assigns an importance weight to each channel of the input feature map. For an input feature map FFF, CAM first compresses spatial information using global average pooling (GAP) and global max pooling (GMP), producing two channel descriptors, $F_{avg}^{c}\;and\;F_{max}^{c}$. These descriptors are then forwarded to a shared two-layer MLP, generating two channel-wise responses. The two responses are fused by element-wise summation and activated by a Sigmoid function to obtain the channel attention map $M_{c}(F)$. Finally, $M_{c}(F)$ is used to rescale the original feature map via element-wise multiplication, resulting in the channel-enhanced feature map $F^{\prime \prime }$.

The channel attention operation can be formulated as follows:

(2) $$\;M_{c}(F)=\sigma (MLP(AvgPool(F))+MLP(MaxPool(F)))=\sigma (W_{1}(W_{0}(F_{avg}^{c}))+W_{1}(W_{0}(F_{max}^{c}))),$$

(3) $$\begin{array}{c} F^{\prime }=M_{c}(F)⊗F, \end{array}$$

In Equations (2) and (3), σ denotes the Sigmoid function, and $W_{0}$ and $W_{1}$ represent the two layers of the shared multilayer perceptron (MLP). $F_{avg}^{c}\;and\;{\;F}_{max}^{c}$ are the channel descriptors obtained via global average pooling and global max pooling, respectively. $M_{c}(F)$ is the resulting channel attention map, $F^{\prime }$ is the feature map after channel-wise recalibration, and ⊗ indicates element-wise multiplication.

Following channel refinement, CBAM employs the Spatial Attention Module (SAM) to further highlight informative spatial regions. Given the feature map processed by CAM, SAM performs average pooling and max pooling along the channel dimension to produce two single-channel feature maps, $\left[F_{avg}^{s};F_{max}^{s}\right]$. These two maps are concatenated to form a fused representation$\;[F_{avg}^{s};F_{max}^{s}]$, which is then fed into a convolution layer with a 7 × 7 kernel. The convolution output is activated by Sigmoid to obtain the spatial attention map. Finally, the spatial attention map is applied to the input feature map through Hadamard (element-wise) multiplication, producing the spatially reweighted output. The spatial attention operation is formulated as follows:

(4) $$\begin{array}{c} M_{s}(F^{\prime })=\sigma (F^{7\times 7}([AvgPool(F^{\prime });MaxPool(F^{\prime })]))\\ \;\;\;=\sigma (F^{7\times 7}([F_{avg}^{s};F_{max}^{s}])) \end{array}$$

(5) $$\begin{array}{cc} F^{\prime \prime } & =M_{s}(F^{\prime })⊗F^{\prime } \end{array}$$

Here, ${\;F}^{7\times 7}$ denotes a convolution operator with a 7 × 7 kernel. $F_{avg}^{s}$ and $F_{max}^{s}$ are the two spatial maps obtained from average pooling and max pooling over the channel dimension in SAM, respectively. The notation $\left[F_{avg}^{s};F_{max}^{s}\right]$ indicates channel-wise concatenation of these two maps. $M_{s}(F^{\prime })$ denotes the spatial attention map computed from $F^{\prime }$.

#### 2.3.3. DyHead Detection Head

The detection head of YOLOv11 adopts a decoupled design and is further optimized under an anchor-free mechanism, which enhances the model’s robustness and adaptability across different detection tasks. Compared with earlier versions, this structure exhibits improved stability in scenarios involving complex backgrounds, large-scale variations, and dense targets. However, in transmission-line defect detection, where multi-scale small objects and background interference are prevalent, there remains room for further performance improvement. To address these challenges, this study introduces the Dynamic Head (DyHead) module [35].

**Figure 7:**
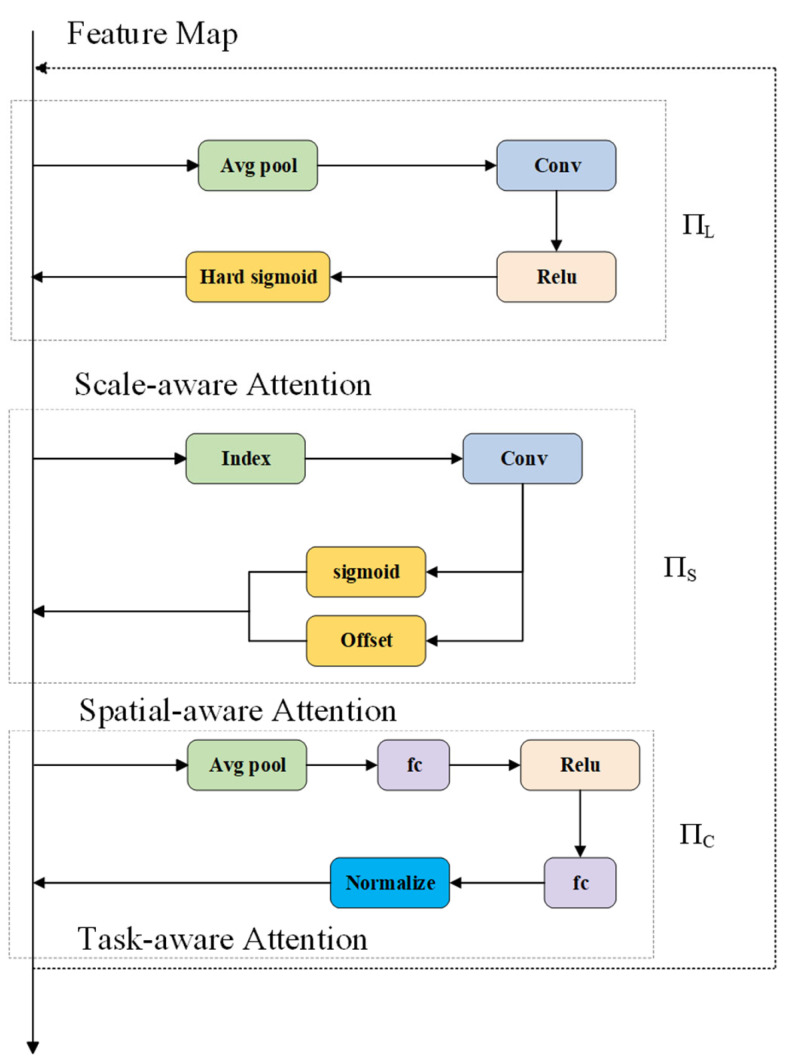
Structure of DyHead detection head network.

DyHead introduces a multi-dimensional attention scheme to enhance feature modeling in the detection head by jointly exploiting scale-related, spatial, and semantic cues, while keeping the additional computation modest. In practice, attention is applied along different dimensions of the feature tensor so that information from feature levels (scales), spatial locations, and channels can be adaptively reweighted and fused. As illustrated in Figure 7, the level/scale-aware branch ($\pi_{L}$) first emphasizes informative feature levels to better accommodate target scale variations. The spatial-aware branch ($\pi_{S}$) then highlights key locations by strengthening position-sensitive responses, allowing the head to focus on regions more likely to contain foreground objects. Finally, the channel/semantic-aware branch ($\pi_{C}$) adjusts channel responses to improve discriminability for detection. With these adaptively modulated features, the detection head generates the final predictions, which is particularly beneficial when defects are small or densely distributed. For a three-dimensional feature tensor $F\in R^{L.\times S.\;\times C.}$, L denotes the feature level (scale) dimension, S denotes the spatial dimension (locations), and C represents the channel dimension.

The spatial attention function is computed as follows:

(6) $$W\left(F\right)\&=\pi_{C}\left(\pi_{S}\left(\pi_{L}\left(F\right)\cdot F\right)\cdot F\right)\cdot F$$

#### 2.3.4. WIoU Loss Function

**Figure 8:**
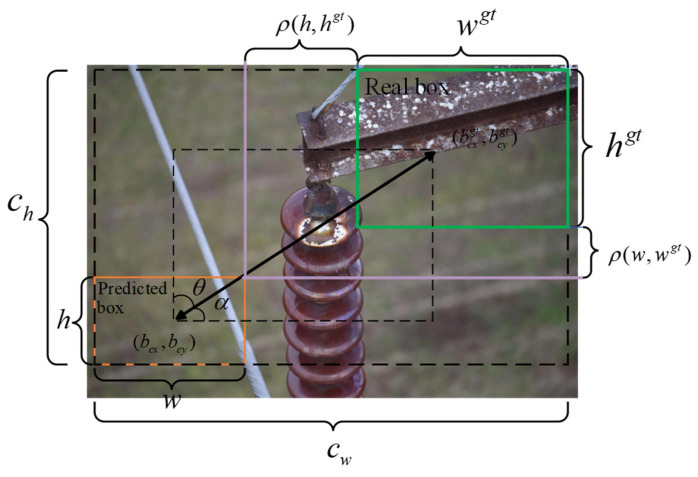
Schematic diagram of loss function parameters.

The bounding box regression loss is crucial in object detection and significantly affects overall model performance. Among various regression metrics, the Intersection over Union (IoU) is commonly used to quantify the overlap between the predicted bounding box and the ground-truth annotation. The geometric definition of IoU is illustrated in Figure 8.

In UAV-based scenarios involving small object detection, the original algorithm employs DFL and CIoU to compute the bounding box regression loss. However, the CIoU function fails to account for the balance between hard and easy samples within the dataset. In contrast, the WIoU loss function enhances the model’s localization capability in classification and regression tasks. Therefore, this study adopts the optimized WIoUv3 [36] to replace CIoU, achieving more accurate bounding box regression performance. Based on distance metric construction, the two-level attention mechanism of WIoUv1 is formulated as follows (Equations (7)–(9)):

(7) $$L_{wiouv1}=R_{wiou}\times L_{iou}$$

(8) $$L_{iou}=1-Iou$$

(9) $$R_{wiou}=exp\left(\frac{{\left(b_{c_{x}}^{g^{t}}-b_{c_{x}}\right)}^{2}+{\left(b_{c_{y}}^{g^{t}}-b_{c_{y}}\right)}^{2}}{\left(c_{w}^{2}+c_{h}^{2}\right)}\right)$$

where ${\;b}_{c_{x}}^{g^{t}}\;and{\;b}_{c_{y}}^{g^{t}}$ denote the coordinates of the center point of the prediction box; $b_{c_{x}}\;and\;b_{c_{y}}$ indicate the coordinates of the center point of the solid box; and $c_{w}\;and\;{\;c}_{h}$ indicate the width and height of the smallest surrounding rectangle between the prediction box and the actual box.

WIoUv3 extends the WIoU formulation by introducing an outlier indicator *β* to represent the quality of anchor boxes. Based on *β*, a non-monotonic focusing term r is derived and incorporated into WIoUv1, resulting in the dynamic non-monotonic focusing mechanism (FM) employed in WIoUv3. This design allows adaptive allocation of gradient gain, enabling medium- and low-quality anchors to receive more appropriate weighting, thereby enhancing the stability of bounding-box regression. The computation of WIoUv3 is expressed as follows:

(10) $$L_{wiouv3}=r\times L_{wiouv1}$$

(11) $$r=\frac{\beta }{\delta \alpha^{\beta -\delta }}$$

(12) $$\beta =\frac{L_{lou}^{*}}{L_{lou}}$$

WIoUv3 further develops the focusing scheme by employing a dynamic coefficient to reweight the bounding-box regression loss during training, thereby enabling more adaptive optimization. This dynamic reweighting mechanism is beneficial to overall detection quality, and it is especially helpful for small-object scenarios commonly encountered in aerial imagery.

### 2.4. Evaluation Metrics

To assess the performance of the proposed method in defect detection, a set of standard evaluation metrics is employed, including Precision, Recall, mAP@0.5, the number of model parameters (Params), and computational cost in GFLOPs. Collectively, these metrics provide a measure of detection accuracy, computational efficiency, and model complexity. Precision and Recall are defined as follows:

(13) $$Precision=\frac{TP}{TP\;+\;FP}$$

(14) $$Recall=\frac{TP}{TP+FN}$$

In Equations (13) and (14), *TP* refers to the number of correctly predicted positive instances (true positives), *FP* counts the cases where negative instances are mistakenly predicted as positive (false positives), and *FN* counts the cases where positive instances are missed and predicted as negative (false negatives). *Precision* quantifies the correctness of positive predictions by measuring the fraction of true positives among all predicted positives, while *Recall* evaluates the coverage of positives by measuring the fraction of detected positives among all ground-truth positives.

The mean Average Precision (*mAP*) is computed as follows:

(15) $$\;\;mAP=\frac{\sum_{i=1}^{N}\;AP_{i}}{N}$$

In this work, *N* denotes the total number of categories in the dataset (*N* = 4), and *APi* is the average precision for the iii-th category. AP summarizes detection performance for a single class, and mAP is obtained by averaging APi over all classes. Specifically, mAP@0.5 (mAP50) is computed at an IoU threshold of 0.50, whereas mAP@0.5:0.95 (mAP50:95) averages the results over multiple IoU thresholds from 0.50 to 0.95 in increments of 0.05.

### 2.5. Test Environment and Parameter Configuration

All experiments were conducted on a Windows operating system using Spyder 5.3.3 with the following environment: PyTorch 2.0.0, Python 3.8, and CUDA 12.1. The hardware environment and model parameters are detailed in Table 2 and Table 3.

## 3. Results

### 3.1. Model Training Results

**Figure 9:**
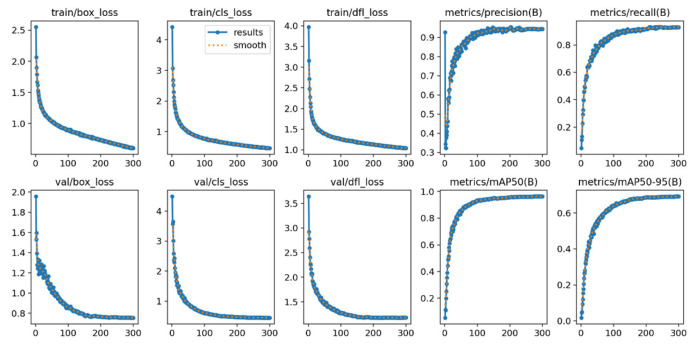
Variation curve of each parameter during training.

To validate the performance of the proposed DCDW-YOLOv11 model for defect detection of key transmission-line equipment, experiments were conducted on the self-constructed transmission-line dataset. Figure 9 presents the evolution of training and validation statistics over epochs. The loss curves (box_loss, cls_loss, and dfl_loss) are reported for both the training and validation sets. All loss terms decrease rapidly at the early stage and then gradually stabilize, indicating stable convergence of the optimization process. In addition, the metric curves show that Precision and Recall increase steadily and plateau toward the end. The mAP@0.5 and mAP@0.5:0.95 curves exhibit similar trends, reflecting consistent performance improvement as training proceeds.

### 3.2. Comparative Experimental Results and Analysis

#### 3.2.1. Comparison of Different Loss Functions

To comprehensively evaluate the applicability and performance of different bounding box regression loss functions in key transmission-line equipment defect detection, a series of comparative experiments were conducted based on the YOLOv11 model. Under identical training configurations and hyperparameter settings, only the regression loss function was replaced. Model performance was evaluated from three perspectives: Precision, Recall, and mean Average Precision (mAP@0.5). The experimental results are summarized in Table 4.

As shown in Table 4, WIoU v3 achieves the best overall performance across all evaluation metrics, with a Precision of 94.4%, a Recall of 92.8%, and an mAP@0.5 of 96.3%, outperforming all other compared loss functions. Although the conventional CIoU loss is widely used in general object detection tasks, it exhibits relatively weaker performance in this study, achieving an mAP@0.5 of 93.4% and a Recall of 87.8%. Both Inner-IoU and DIoU demonstrate competitive detection performance, with Inner-IoU reaching a Recall of 92.3% and an mAP@0.5 of 95.5%, but still slightly inferior to WIoU v3. Notably, by introducing a dynamic weighting strategy, WIoU v3 places greater emphasis on high-quality bounding box regression, thereby enhancing the model’s sensitivity to critical target regions and improving localization accuracy. Moreover, WIoU v3 exhibits strong robustness in recognizing multi-scale defect targets under complex background conditions, indicating favorable generalization capability and resistance to background interference.

#### 3.2.2. Comparison of Different Attention Mechanisms

To further verify the effectiveness of attention mechanisms in key transmission-line equipment defect detection, comparative experiments were conducted by integrating several representative attention modules into a unified YOLOv11-based architecture under identical training settings. The evaluated attention mechanisms include SE, SimAM, CPCA, EMA, and CBAM. The experimental results are presented in Table 5. As shown, CBAM achieves the highest performance across all evaluation metrics, with a Precision of 94.4%, Recall of 92.8%, and mAP@0.5 of 96.3%. Compared to the other attention mechanisms, CBAM not only improves detection accuracy but also substantially enhances recall, indicating its ability to more comprehensively focus on defect regions, effectively reduce missed detections, and strengthen overall detection performance in complex inspection scenarios. The performance of different attention mechanisms exhibits distinct characteristics. The SE module enhances feature representation by modeling channel-wise dependencies and achieves competitive precision; however, the absence of spatial attention results in a relatively lower recall, limiting its ability to fully capture defect regions. The lightweight SimAM module offers high computational efficiency but shows insufficient detection accuracy in this task. CPCA strengthens cross-position channel coupling and improves spatial awareness; however, its overall results are still slightly weaker than those achieved by CBAM. EMA is able to aggregate multi-scale contextual cues, which is helpful for handling defects with different sizes, yet it may not always attend to the most informative regions with sufficient precision. By contrast, CBAM combines channel-wise and spatial-wise attention in a unified manner, allowing the network to highlight defect-relevant responses while reducing interference from complex backgrounds. This coupled attention design improves feature separability and yields more complete semantic representations. In practical transmission-line inspection, where defect appearances vary considerably and the background is often cluttered, CBAM exhibits stronger robustness and adaptability. As a result, CBAM contributes to higher detection accuracy and recall, and it also supports better generalization and more stable performance under challenging conditions.

#### 3.2.3. Performance Comparison with Different Detection Models

To comprehensively assess the effectiveness of the proposed method, several representative object detection algorithms were chosen as baseline models, including Faster R-CNN, YOLOv5, YOLOv8, YOLOv10, and YOLOv11. All models were trained and evaluated under identical experimental settings on the constructed key transmission-line equipment defect dataset. The quantitative comparison results are summarized in Table 6. As shown in Table 6, the two-stage detector Faster R-CNN exhibits strong feature extraction capability; however, its large number of parameters and high floating-point computational cost result in a bulky model size and relatively lower detection accuracy. These limitations hinder its applicability in scenarios requiring lightweight deployment and real-time inference, thereby reducing its practicality for rapid transmission-line defect detection tasks. In contrast, the YOLO family, as representative one-stage detectors, demonstrates superior lightweight characteristics and real-time performance. On this basis, the proposed DCDW-YOLOv11 consistently outperforms the compared YOLO-based models across multiple key evaluation metrics. Specifically, in terms of precision, DCDW-YOLOv11 achieves improvements of 15.9%, 2.3%, 0.9%, 11.5%, and 2.8% over Faster R-CNN, YOLOv5, YOLOv8, YOLOv10, and YOLOv11, respectively. For recall, the corresponding improvements are 20.5%, 7.5%, 4.3%, 15.0%, and 7.0%, indicating a notable advantage in enhancing detection completeness and reducing missed detections. Moreover, in terms of mAP@0.5, DCDW-YOLOv11 surpasses the above models by 16.2%, 4.2%, 2.8%, 10.9%, and 4.31%, respectively, further validating its superior detection accuracy.

Overall, these results demonstrate that DCDW-YOLOv11 achieves a more favorable balance between detection accuracy and robustness while maintaining the efficiency advantages of one-stage detectors, making it particularly well-suited for key transmission-line equipment defect detection in complex field environments.

### 3.3. Ablation Study

To quantitatively evaluate the contributions of different components in the proposed DCDW-YOLOv11 framework, a stage-wise ablation study was conducted using the official YOLOv11 model as the baseline. Considering that the proposed improvements are designed to operate cooperatively at different stages of the detection pipeline, the ablation experiments were organized following an incremental and modular integration strategy. Representative module combinations were progressively introduced while keeping all other training settings unchanged. The corresponding results are summarized in Table 7.

The baseline YOLOv11 model achieves a Precision of 91.9%, a Recall of 87.8%, and an mAP@0.5 of 93.4%, with a compact model size of 2.5 MB and 6.3 GFLOPs, indicating that the baseline is lightweight but exhibits limited capability in handling complex backgrounds and small-scale defects.

First, the C3K2-DCNv3 module was introduced into the backbone network. This modification leads to consistent performance improvements, with Precision, Recall, and mAP@0.5 increasing to 92.7%, 90.1%, and 94.0%, respectively, while maintaining nearly identical computational complexity. The observed gain, particularly in Recall, demonstrates that deformable convolution effectively enhances the model’s ability to capture geometric variations and irregular defect patterns, thereby reducing missed detections in complex inspection scenarios.

Next, the CBAM attention mechanism was integrated together with C3K2-DCNv3 to further refine feature representation. As shown in Table 7, this combination improves Precision and Recall to 93.0% and 91.1%, respectively, with mAP@0.5 reaching 94.8%. By jointly modeling channel-wise and spatial attention, CBAM guides the network to focus on discriminative defect regions while suppressing background interference, resulting in more robust and informative features.

Based on the above configuration, the original bounding box regression loss was replaced by the WIoU loss function. This modification further improves localization quality, yielding an mAP@0.5 of 95.0% without increasing model parameters or FLOPs. This improvement indicates that WIoU effectively emphasizes high-quality predictions through dynamic sample reweighting, thereby enhancing bounding box regression accuracy, especially for small-scale and ambiguous defects.

Finally, the DyHead dynamic detection head was incorporated to strengthen multi-scale feature fusion and task-aware representation learning. With all modules enabled, the complete DCDW-YOLOv11 model achieves the best overall performance, reaching 94.4% Precision, 92.8% Recall, and 96.3% mAP@0.5. Although the model size and computational cost slightly increase to 3.0 MB and 7.4 GFLOPs, the performance gain is substantial, confirming the effectiveness of DyHead in handling multi-scale defects under complex background conditions.

Overall, the ablation results demonstrate that the proposed modules contribute to performance improvements at different stages of the detection pipeline. Rather than acting independently, these components exhibit strong complementarity, and their joint integration yields a clear synergistic effect, leading to significant enhancements in detection accuracy and robustness while preserving favorable lightweight characteristics. This systematic ablation analysis validates the rationality of the proposed DCDW-YOLOv11 design for key transmission-line equipment defect detection in real-world UAV inspection scenarios.

**Figure 10:**
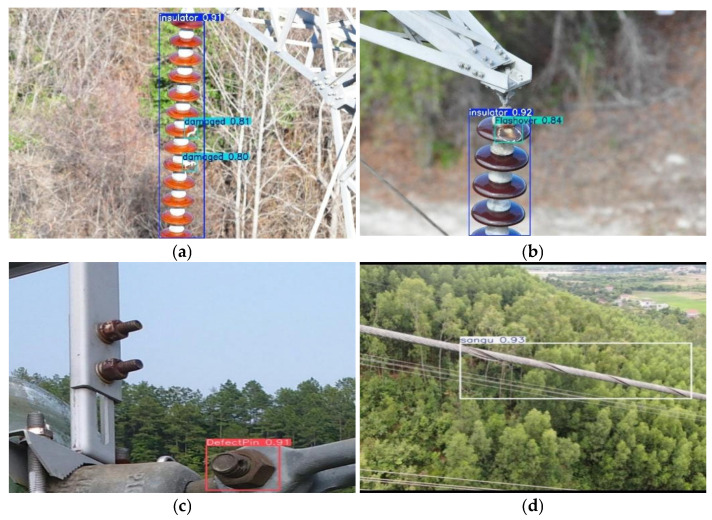
Detection results of key equipment defects in DCDW-YOLOv11 transmission lines. (**a**) Damaged. (**b**) Flashover. (**c**) DefectPin. (**d**) Sangu.

Figure 10 further presents qualitative detection results of DCDW-YOLOv11 on real-world transmission-line images, visually confirming its effectiveness in identifying multi-scale and multi-type defects under challenging conditions. In addition, Table 8 summarizes the detection performance across representative defect categories, where DCDW-YOLOv11 consistently achieves superior accuracy, demonstrating strong robustness, generalization capability, and practical applicability in complex engineering environments.

### 3.4. Model Validation and Visualization Analysis

#### 3.4.1. Algorithm Validation

**Figure 11:**
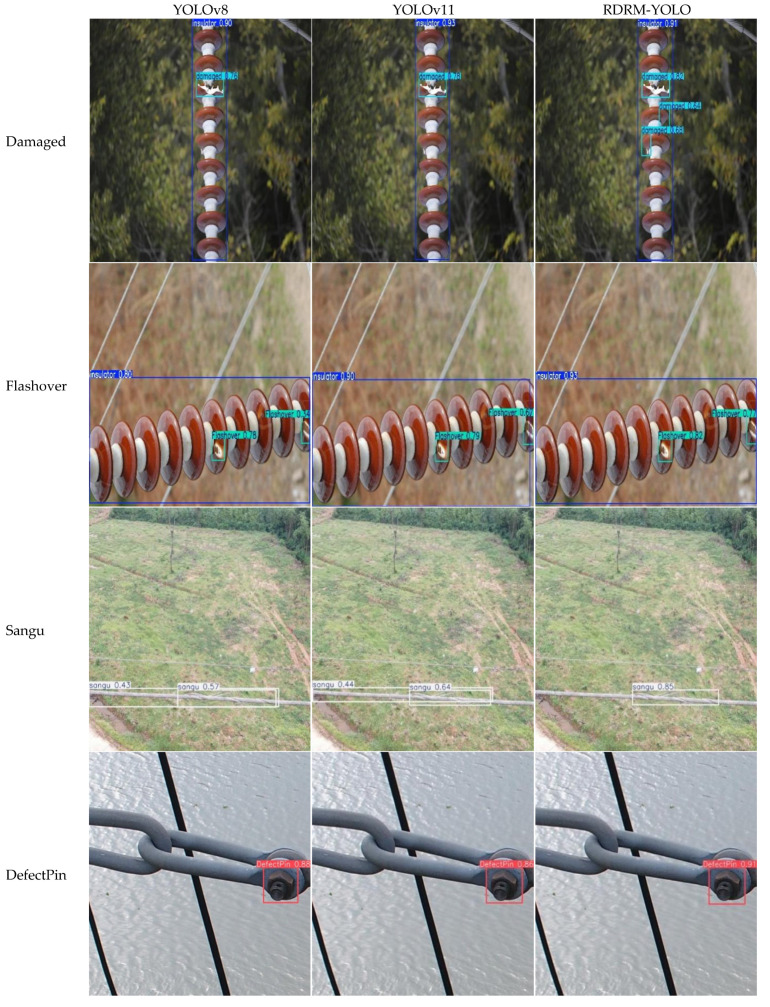
The visualization effect of different models on identifying key equipment defects.

To provide a more intuitive validation and comparative analysis of the effectiveness of the proposed DCDW-YOLOv11 model in defect detection tasks, two high-performing baseline models, YOLOv8 and YOLOv11, were selected for comparison. Representative test images containing various types of defects were randomly sampled from the test set, and qualitative detection results were visualized and compared, as shown in Figure 11. As observed in Figure 11, both YOLOv8 and YOLOv11 exhibit a certain degree of missed detections when handling complex scenes, particularly in images with densely distributed multiple targets, where detection results become unstable and target omissions frequently occur. In addition, false detections are observed in images containing specific defect types (e.g., conductor strand breakage), indicating that these models still suffer from limitations in multi-scale target perception and feature representation.

In contrast, the improved DCDW-YOLOv11 model demonstrates significantly superior detection performance. It achieves more accurate localization and classification across different defect categories and effectively adapts to defects with varying scales and morphological characteristics, thereby substantially reducing both missed detections and false positives. Overall, the qualitative results indicate that DCDW-YOLOv11 exhibits strong robustness and generalization capability across all four categories of key equipment defects, confirming its effectiveness in complex inspection scenarios.

#### 3.4.2. Feature Visualization Analysis

**Figure 12:**
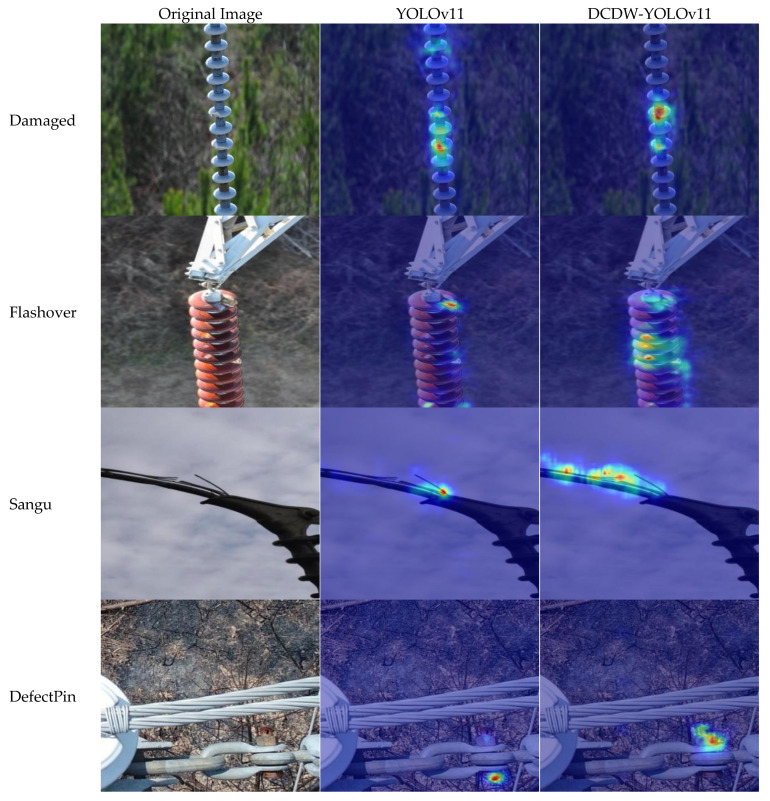
Model heat map visualization.

To further evaluate the proposed model’s ability to focus on defect regions, LayerCAM [45] was employed for feature visualization analysis. Figure 12 presents the heatmap distributions generated by the model when processing representative defect images. In these visualizations, color intensity indicates the degree of attention assigned by the model to different regions, where darker colors correspond to regions that contribute more significantly to the final prediction.

Figure 12 present a comparative visualization of the heatmap results generated by YOLOv11 and the proposed DCDW-YOLOv11 model for key transmission-line equipment defect detection. These visualizations clearly reveal notable differences in target attention behavior and feature discrimination mechanisms between the two models. Compared with YOLOv11, DCDW-YOLOv11 exhibits higher response intensity and more spatially concentrated activation within defect regions, indicating a stronger focus on critical defect features and enhanced sensitivity in feature representation. In contrast, YOLOv11 shows evident response drift in certain scenarios, with activation regions partially concentrated on non-defect areas. This behavior increases the likelihood of misinterpreting background structures or non-target components as defect targets, highlighting limitations in target discrimination and precise localization under complex background conditions. From a structural perspective, the performance improvement of DCDW-YOLOv11 can be attributed to the systematic integration of the C3K2-DCNv3 module, CBAM attention mechanism, WIoU loss function, and DyHead detection head. On one hand, deformable convolution combined with multi-scale feature aggregation enhances the model’s adaptability to geometric deformations and scale variations, thereby improving its ability to extract fine-grained defect features and capture semantic information. On the feature-extraction side, combining deformable convolution with multi-scale feature integration enables the network to better accommodate geometric distortions and scale changes, which helps preserve subtle defect details and enrich semantic representation. On the optimization side, the coupled channel–spatial attention together with the reweighted loss formulation reinforces responses in informative regions while attenuating background-driven activations. As a consequence, attention becomes more concentrated on genuine defect areas, false alarms are reduced, and overall detection accuracy and robustness are improved. For the Flashover defect, DCDW-YOLOv11 generates heatmaps that respond to a wider set of abnormal cues, allowing the model to capture subtle variations in texture and brightness around discharge regions more sensitively. This indicates enhanced stability and adaptability when dealing with fine-grained defects under complex backgrounds. In the DefectPin task, the proposed method not only highlights all true defect regions, but also avoids the spurious activations on nearby non-defective structures that are observed in YOLOv11, demonstrating stronger target discrimination and more reliable decision making.

#### 3.4.3. Robustness Analysis Under Rainy Conditions

To further evaluate the robustness of the proposed DCDW-YOLOv11 under rainy visual interference conditions, qualitative experiments were conducted using synthetically generated rainy images. Specifically, drawing on relevant methodologies [46,47,48], we introduced rain-induced visual degradations into our self-built dataset to simulate common imaging artifacts encountered during rainy-day inspections of transmission lines. These artifacts include rain streak noise, reduced contrast, and local occlusions.

**Figure 13:**
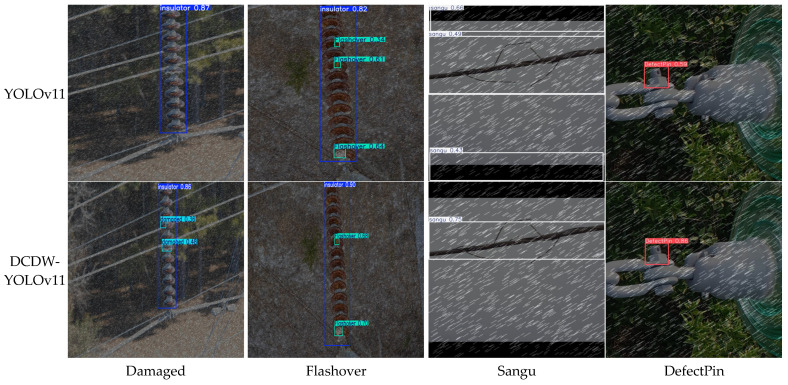
Qualitative comparison of defect detection results under rainy conditions.

Figure 13 illustrates representative detection results for four typical defect categories, namely Damaged, Flashover, Sangu, and DefectPin, under rainy conditions. It can be observed that the baseline YOLOv11 model suffers from noticeable performance degradation in the presence of rainy visual interference, which is mainly reflected in an increased number of missed detections, degraded localization accuracy, and reduced detection confidence. These issues are particularly evident for small-scale and low-contrast defects.

In contrast, the proposed DCDW-YOLOv11 demonstrates more stable and reliable detection performance across all four defect categories. Even under rainy visual interference, the model is able to maintain clearer defect localization results and relatively higher detection confidence. This performance advantage is primarily attributed to the enhanced feature representation capability of DCDW-YOLOv11, which enables effective discrimination of defect-related features under degraded imaging conditions.

It should be emphasized that the rainy images used in this study are synthetically generated and are intended to serve as a supplementary robustness evaluation, rather than a complete substitute for data collected under real-world adverse weather conditions. Nevertheless, the qualitative results provide useful evidence that DCDW-YOLOv11 exhibits superior stability and generalization capability compared with YOLOv11 when confronted with rain-induced visual interference.

## 4. Discussion

### 4.1. Generalization Ability in Practical Transmission-Line Inspection

Generalization ability is a critical requirement for defect detection models deployed in real-world transmission-line inspection scenarios. In this study, all data were collected using similar UAV platforms and inspection procedures. It should be noted that transmission-line equipment follows unified national and industry standards, resulting in relatively limited structural variation across different inspection regions.

Compared with natural-scene object detection tasks, the domain shift caused by structural variation is relatively constrained in transmission-line inspection. Instead, the primary challenges to generalization arise from complex backgrounds, variations in defect scale, and viewpoint diversity. These factors are representative of practical inspection conditions and constitute the main sources of performance degradation in UAV-based inspection scenarios.

To address these challenges, the proposed DCDW-YOLOv11 model strengthens robust feature learning under varying defect scales and enhances background suppression capability. Through task-oriented architectural optimization, the model maintains stable detection performance across diverse inspection images within the same standardized infrastructure domain under complex background conditions.

Most existing studies on transmission-line defect detection rely on standard convolutional backbones or fixed-structure detection heads [49], which may limit adaptability to background interference and scale variation commonly encountered in UAV-based inspection. In contrast, DCDW-YOLOv11 introduces deformable convolution and dynamic detection head mechanisms, enabling more flexible feature modeling and improved robustness in complex inspection environments.

### 4.2. Dataset Bias and Class Imbalance

In practical transmission-line inspection, the occurrence frequency of different defect types is inherently imbalanced. Certain defect categories appear more frequently during routine inspections, while others occur less often but may pose higher operational risks. This imbalance can bias the training process toward dominant categories and adversely affect detection reliability for underrepresented defects.

In this work, dataset bias is partially alleviated through data augmentation strategies that increase sample diversity and reduce overfitting to frequently observed defect types. Moreover, the dataset was collected across multiple cities and inspection environments, introducing variations in background complexity and viewing angles. Such intra-domain diversity contributes to improved model robustness within the same application context, although imbalance-related challenges remain.

Compared with several existing UAV-based inspection approaches reported in the literature [50], the proposed method demonstrates a more balanced improvement in precision and recall. This balanced performance is particularly important in practical inspection tasks, where both missed detections and false alarms may lead to increased operational risks or unnecessary maintenance costs.

### 4.3. Effect of Model Design on Bias Mitigation

Beyond data-level strategies, the architectural and optimization choices adopted in DCDW-YOLOv11 contribute to mitigating the negative effects of dataset bias. The employed dynamic detection head enhances feature fusion across different feature levels, enabling more reliable detection under defect scale variation. In addition, attention-guided feature extraction encourages the network to focus on defect-relevant regions while suppressing background interference, which is beneficial for detecting visually subtle defect patterns.

Furthermore, the WIoU-based loss function introduces adaptive optimization behavior by reducing the dominance of easy samples during training and promoting learning from harder examples. This mechanism improves localization accuracy and classification stability, particularly for defect categories with limited representation in the dataset, without relying on explicit re-sampling or class re-weighting techniques.

### 4.4. Limitations and Future Directions

Despite the encouraging performance achieved by the proposed DCDW-YOLOv11 model, several limitations should be acknowledged. First, although the standardized design of transmission-line equipment results in relatively limited structural variation across different regions, the experimental evaluation in this study is mainly conducted within a single application domain. Therefore, further validation across more diverse inspection platforms and operational conditions is required to provide a more comprehensive assessment of the model’s robustness.

Second, the dataset used in this work exhibits an imbalanced distribution of defect categories, which reflects the inherent characteristics of real-world transmission-line inspection data. Although the adopted architectural design and optimization strategies partially mitigate the impact of data imbalance, the detection reliability of individual defect categories under unseen conditions may still be affected.

Moreover, the experimental analysis under adverse weather conditions in this study is primarily based on synthetically generated data. While such data offer a controllable and reproducible means for robustness evaluation, they cannot fully capture the complexity and variability of real-world weather environments.

Looking ahead, future research will focus on extending model validation to more diverse inspection scenarios, particularly through the collection and utilization of real-world datasets acquired under complex weather conditions, such as rain, fog, and low-visibility environments. In parallel, imbalance-aware learning strategies will be further investigated to enhance the generalization capability of the proposed model. Additionally, lightweight model design remains a critical research direction for large-scale UAV-based deployment. Potential efforts include the exploration of more efficient convolutional operators, structured model compression techniques such as pruning and knowledge distillation, as well as deployment-aware inference strategies that adapt computational complexity to varying scene characteristics. These directions are expected to improve real-time performance and operational efficiency while maintaining high detection accuracy.

## 5. Conclusions

In this study, a task-oriented and system-level optimized YOLOv11-based defect detection framework, termed DCDW-YOLOv11, is proposed for UAV-based transmission-line inspection. By integrating the C3K2-DCNv3 module and CBAM attention mechanism into the backbone network, adopting the DyHead dynamic detection head, and employing the WIoU loss function for bounding box regression, the proposed model achieves coordinated improvements in feature representation, small-scale defect detection, and robustness under complex background conditions.

Experimental results on the self-constructed transmission-line defect dataset demonstrate that DCDW-YOLOv11 achieves an mAP@0.5 of 96.3%, a Precision of 94.4%, and a Recall of 92.8%, outperforming the baseline YOLOv11 model by 2.8, 7.0, and 4.4 percentage points, respectively. These results confirm that the effective integration of complementary architectural and optimization strategies can significantly enhance detection reliability in practical UAV inspection scenarios.

Rather than introducing a fundamentally new detection paradigm, this work emphasizes an engineering-oriented design that tailors existing advanced techniques to the specific requirements of transmission-line inspection. From an application perspective, the achieved balance between accuracy and robustness satisfies the operational demands of real-world UAV-based inspection tasks.

Although the experiments were conducted on a self-constructed dataset, the data were collected across multiple cities and inspection environments, providing an initial validation of model robustness within the same application domain. Future work will focus on extending experimental validation to more diverse inspection scenarios, further improving model generalization, and optimizing inference efficiency to better support large-scale and real-time UAV deployment.

## Figures and Tables

**Table 1:** Composition of defect dataset for key transmission-line equipment.

Defects in Key Transmission-Line Equipment	DefectPin	Insulator		Sangu
		Damaged	Flashover	
Total Number of Samples	1209	867	822	2372
Number of Training Sets	967	693	657	1897
Number of Validation Sets	242	174	165	475

**Table 2:** Experimental environment configuration.

Configuration Items	Configuration Parameters
GPUs	NVIDIA RTX A4000
CPU	Intel(R) Xeon(R) W5-2455X
CUDA	12.1
RAM	16 GB
Computer operating system	Windows 11

**Table 3:** Model parameter settings.

Training Paramars	Values
Optimizer	SGD
Input image size	640 × 640
Initial learning rate	0.01
Optimizer momentum	0.937
Optimizer weight decay rate	0.0005
Number of images per batch	32
Patience	100
Number of epochs	300

**Table 4:** Comparative analysis of different loss functions.

Metrics	Precision/%	Recall/%	mAP@0.5/%
CIoU	91.9	87.8	93.4
DIoU [37]	93.7	90.0	94.1
GIoU [38]	90.9	86.4	91.7
SIoU [39]	91.8	891	93.8
Inner-IoU [40]	93.5	92.3	95.5
WIoU v3	94.4	92.8	96.3

**Table 5:** Comparative analysis of different attention mechanisms.

Attention Mechanisms	Precision/%	Recall/%	mAP@0.5/%
SE [41]	93.7	91.9	95.3
SimAM [42]	94.1	90.3	94.7
CPCA [43]	92.7	90.1	94.0
EMA [44]	93.1	91.1	94.9
CBAM	94.4	92.8	96.3

**Table 6:** Contrast experiment.

Models	Precision/%	Recall/%	mAP@0.5/%	FLOPs/G	Parameters/MB	Model Size/MB
Faster R-CNN	84.1	72.3	80.1	71.6	41.4	321.0
YOLOv5	92.1	85.3	92.1	7.1	2.5	5.1
YOLOv8	93.5	88.5	93.5	8.1	3.0	6.0
YOLOv10	82.9	77.8	85.4	6.5	2.2	5.5
YOLOv11	91.6	85.8	91.9	6.3	2.5	5.3
DCDW-YOLOv11	94.4	92.8	96.3	7.4	3.0	6.2

**Table 7:** Ablation experiment.

C3K2- DCNv3	CBAM	WIoU	DyHead	Parameters/MB	FLOPs/G	Precision/%	Recall/%	mAP@0.5/%
				2.5	6.3	91.9	87.8	93.4
✓				2.5	6.2	92.7	90.1	94.0
	✓			2.5	6.2	92.4	89.3	93.8
		✓		2.5	6.2	92.1	89.0	93.7
			✓	3.0	7.4	93.0	90.6	94.7
✓	✓			2.5	6.2	93.0	91.1	94.8
✓	✓	✓		2.5	6.2	93.1	91.8	95.0
✓	✓	✓	✓	3.0	7.4	94.4	92.8	96.3

Note: ‘✓’ indicates that this module or feature is included in the model during experimentation.

**Table 8:** Comparison of performance parameters for different defects.

Defects in Transmission-Line Equipment	Precision/%	Recall/%	mAP@0.5/%
Damaged	94.3	90.5	93.5
Flashover	91.9	83.3	91.2
DefectPin	90.1	86.9	94.8
Sangu	96.1	98.4	99.0

## Data Availability

The original contributions presented in this study are included in the article. Further inquiries can be directed to the corresponding author(s).
